# Pd-Free Activation Pretreatment for Electroless Ni-P Plating on NiFe_2_O_4_ Particles

**DOI:** 10.3390/ma11101810

**Published:** 2018-09-24

**Authors:** Junfei Ma, Zhigang Zhang, Yihan Liu, Xiao Zhang, Hongjie Luo, Guangchun Yao

**Affiliations:** School of Metallurgy, Northeastern University, Shenyang 110819, China; mandy0322@163.com (J.M.); liuyh@smm.neu.edu.cn (Y.L.); 13609882383@126.com (X.Z.); luohj@smm.neu.edu.cn (H.L.); yaogc@smm.neu.edu.cn (G.Y.)

**Keywords:** electroless plating, NiFe_2_O_4_, activation, microstructure

## Abstract

A Pd-free activation pretreatment process was developed for electroless Ni-P plating on NiFe_2_O_4_ particles. The main influencing factors, including NiCl_2_·6H_2_O concentration, pH of electroless bath and temperature, were investigated. Microstructures of the coating layers were characterized by scanning electron microscopy. It was found that a more uniform and compact Ni-P coating layer was successfully formed by electroless plating via Pd-free activation pretreatment than Pd as sited plating. The coating layers plated by Pd-free activation pretreatment were thicker than those by the sensitization and activation pretreatment on average (9 vs. 5 μm). The new process did not need conventional sensitization or activation pretreatments, because the Ni particles dispersed uniformly on the NiFe_2_O_4_ substrate became catalytic activation sites for nickel electroless plating. Such improvement was beneficial to shortening the preparation process and reducing the production costs with the use of noble metal Pd.

## 1. Introduction

NiFe_2_O_4_ ceramic is a very promising inert anode for aluminum electrolysis owing to its high thermal stability, chemical stability, and high corrosion resistance against molten cryolite [1,2,3,4,5]. However, the low electroconductivity of NiFe_2_O_4_ does not meet the basic requirement [6,7], but can be improved effectively by adding conductive metal [8,9,10]. Metal Ni is the common metallic phase added to inert anode, under the nitrogen protection, Ni after sintering can still be well kept in the matrix [11,12]. Ball mixing and electroless plating are the main methods to add the metallic phase for cermet preparation [13,14,15]. Mechanical mixing, which is operationally simple and short, is mostly adopted to mix metallic and ceramic components to prepare cermet. However, the metallic phase hardly distributes uniformly and easily agglomerates, where the properties are debased dramatically [16,17,18]. A network structure of metallic phase is formed by metal coating on the surface of ceramic particles, where the properties especially conductivity are improved significantly [19,20,21].

In conventional pretreatment of electroless Ni plating, Sn and Pd are usually deposited on the substrate surface by sensitization and activation treatments [22,23,24]. However, these treatments are limited by many problems, such as the use of highly toxic tin, high cost of noble metal, and uncontrollability which results in the waste of Pd and the failure of plating [25,26]. Therefore, finding a new pretreatment without sensitization and activation becomes a research hotspot to improve the electroless plating process. Tang et al. prepared an ABS–CTS–Ni structure in which the Ni nanoparticles became catalytic sites for electroless Ni plating [27]. As reported, the ABS surfaces became rough after etching by H_2_SO_4_-MnO_2_ colloid and were found with newly formed carboxyl and hydroxyl groups after absorption and reduction in a sodium borohydride solution, the ABS surfaces were deposited with copper particles, which replaced the catalyst for SnCl_2_/PdCl_2_ colloid [28]. Moreover, the surface of engineering plastic was treated with an activation solution and supersonic wave to produce surface defects as the active site for the direct electroless copper plating [26]. Li et al. provided a new way to obtain porous MgO film on the surface of AZ91D magnesium alloy by the micro-arc oxidation technology [29]. An electroless plated Ni layer can be prepared on the surface of porous MgO film to improve the surface activity of porous structure. Tian et al. developed a Ni-activation method for electroless Ni deposition on inert copper substrates through negatively shifting copper potential in the presence of high-concentration thiourea in acidic solution [30]. Nobari et al. prepared a seed layer of copper nanoparticles for activating the glass substrate, and found the basic reaction involved copper ion reduction by adding hydrazine hydrate as a reducing agent to the CuSO_4_·5H_2_O and NH_4_OH solution [31]. The above studies suggest catalytic activation sites on the surface matrix for electroless Ni plating without sensitization-activation pretreatment can be prepared. During research on Pd-free treatment, the types of substrates include organics, metals, alloys, and glass, but there is rare report about Pd-free treatment with ceramic substrates.

In this study, a Pd-free activation pretreatment for electroless Ni-P plating was studied. NiFe_2_O_4_ substrates with numerous uniformly dispersed Ni particles, which acted as catalytic activation sites, were prepared by powder metallurgy. Then the NiFe_2_O_4_ substrates were electroless plated with continuous Ni-P coatings. Because of low-cost and environmental friendlies, this novel pretreatment was of great significance and could be applied to large-scale commercial manufacturing. The effects of deposition conditions on the weight gain rate and morphological properties of the Ni-P coatings were also studied.

## 2. Experimental Procedures

Nickel monoxide (NiO ≥ 99.0%), iron oxide (Fe_2_O_3_ ≥ 99.0%), nickel powder (Ni ≥ 99.5%), nickel dichloride hexahydrate (NiCl_2_·6H_2_O ≥ 98.0%), stannous chloride dihydrate (SnCl_2_·2H_2_O ≥ 98.0%), palladium chloride (PdCl_2_ ≥ 99.8%), sodium hypophosphite (NaH_2_PO_2_·H_2_O ≥ 99.0%), trisodium citrate (C_6_H_5_Na_3_O_7_·2H_2_O ≥ 99.0%), sodium acetate (CH_3_COONa ≥ 99.0%), lactic acid (C_3_H_6_O_3_, 85~90%), hydrochloric acid (HCl, 36~38%) and ammonium hydroxide (NH_3_·H_2_O, 25~28%) were purchased from Sinopharm Chemical Reagent Co., Ltd. (Shanghai, China). Chemicals that were used in this study were analytical grade and were used as received without further purification. Double distilled and deionized water was used as a solvent.

### 2.1. Sensitization and Activation Pretreatments

NiFe_2_O_4_ particles were prepared from powder metallurgy. Firstly, NiO and Fe_2_O_3_ powders were mixed, milled in distilled water by ball-milling for 24 h, and then dried thoroughly. The dried mixture was molded by cold pressing under 60 MPa pressure and then air-calcined at 1200 °C in air for 6 h to from NiFe_2_O_4_ spinel matrix. After crushing and screening, NiFe_2_O_4_ particles were obtained by washing away the NiFe_2_O_4_ fine powder attached on the particle surfaces with deionized water.

The as-prepared NiFe_2_O_4_ particles were first sensitized at 60 °C in an aqueous solution containing 10 g/L SnCl_2_ and 10 vol.% HCl, placed until the solution became clear, and then activated in an aqueous solution of 0.5 g/L PdCl_2_ and 10 vol.% HCl for 10 min.

### 2.2. Pd-free Activation Pretreatment

Pd-free activation was performed by directly inlaying Ni particles on the substrate as catalyst sites for electroless Ni-P plating. Ni/NiFe_2_O_4_ cermets were fabricated by cold pressing and sintering proper amounts of Ni (4 wt.%), NiO and Fe_2_O_3_. Through ball-milling and drying, green bodies were molded by cold pressing under 60 MPa pressure and calcined at 1200 °C for 6 h in a nitrogen atmosphere. After crushing and screening, the fine powder on the particle surfaces was washed away with deionized water. Ni-NiFe_2_O_4_ particles were selected for electroless plating.

### 2.3. Electroless Plating

Electroless plating was carried out in air without the protection of nitrogen or inert gas, under simultaneous stirring by a mechanical agitator. The reaction solution was made up of NaH_2_PO_2_·H_2_O, Ni_2_Cl_2_·6H_2_O, Na_3_C_6_H_5_O_7_·2H_2_O and CH_3_COONa. During the reaction, the system was kept at constant pH by adding an NH_3_·H_2_O solution. The load was 30 g/L. Finally, after washing several times with distilled water to remove all impurities, the plated particles were collected by vacuum filtration and then vacuum-dried at 100 °C for 2 h. The substrate particles treated by the two activation methods were electrolessly plated separately under the same plating parameters, summarized in Table 1.

### 2.4. Characterization

Particle sizes and specific surface areas of electroless plating substrate particles were measured using a Bettersize 2000 laser particle size analyzer (Bettersize, Dandong, Liaoning, China). The sizes of particles in the 10% (D10, small particles), 50% (D50, median particle size) and 90% (D90, large particles) of particle size distribution were measured. All results were the average of 5 groups of measurements. The errors of repeatability and accuracy were both ≤0.5%. The microstructures and surface compositions of the sensitized and activated NiFe_2_O_4_ particles, and the Pd-free treated Ni-NiFe_2_O_4_ particles were observed by Ultra Plus field emission scanning electron microscopy (Zeiss, Oberkochen, Baden-Württemberg, Germany) and energy dispersive spectrometry (EDS). Surface roughness (Sa) of the coatings was measured using a LEXT OLS4100 3D measuring laser microscope (Olympus, Tokyo, Japan). Surface morphology and thickness of electroless Ni-P coatings on NiFe_2_O_4_ and Ni/NiFe_2_O_4_ particles were investigated by FESEM and the coating compositions were analyzed by EDS. The weight gain rate *R* of the Ni-P layer after the electroless plating reaction was determined via a weight gain method:

(1) $$R=\frac{m_{2}-m_{1}}{m_{1}}\times 100\%$$

where *m*_2_ is the mass of the object plated after a time duration of *t*, and *m*_1_ is the initial mass of the substrate. All electroless plating reactions took 70 min. Thus, *R* reflected the deposition efficiency of the electroless plating reaction on substrate surfaces in the plating bath. *R* was the average value of 5 measurements since reach electroless plating reaction under each type of conditions was conducted 5 times.

## 3. Results and Discussion

The sizes and specific surface areas of the substrate particles in the electroless plating experiments are shown in Table 2.

As shown in Table 2, the two types of substrate particles were not largely different in median sizes (D50), small-particle distribution (D10), large-particle distribution (D90) or specific surface area, indicating for substrates treated by different activation methods, the loading amounts in the plating solutions were similar when the weight conditions were the same.

### 3.1. Pd Activation and Pd-Free Activation Pretreatment

**Figure 1:**
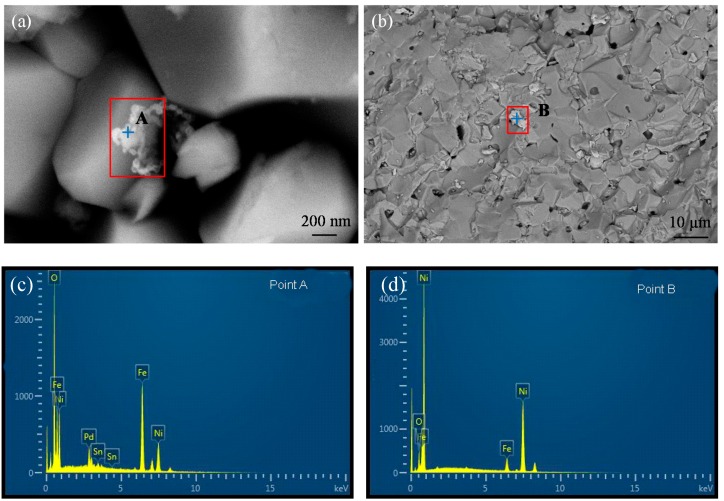
SEM images of (**a**) Pd activated NiFe_2_O_4_ particles, (**b**) Ni-NiFe_2_O_4_ particles, and EDS results of (**c**) at point A, (**d**) at point B.

The electroless Ni reaction is autocatalytic, which means the reaction continues once an initial Ni layer is created. This reaction is so promising that Ni can act successfully as catalyst sites for Ni deposition. In this work, the activation process was performed by directly inlaying Ni particles on the substrate as catalyst sites for electroless Ni-P plating. Since the result of activation would affect the electroless plating, the amounts and distributions of active metals (Pd and Ni) on particle surfaces after different pretreatments were compared, showing in Figure 1.

After sensitization and activation pretreatment (Figure 1a), the Pd was confirmed on top the surface of NiFe_2_O_4_ particle according to the EDS results (Figure 1c) and appeared on the surface of original ceramic grains. However, Pd atoms accumulated at the ‘nm’ scale, but were rarely adsorbed onto particle surfaces (Figure 1a). The peaks of Pd indicated the less quantity of Pd particles. The Pd concentration at the enrichment site was determined by EDS spectra to be 5.55 wt.%.

Figure 1b shows the SEM image of Ni-NiFe_2_O_4_particles produced with the described pretreatment method. The enrichment zone of Ni particles was at the micrometer scale. Together with the EDS results (Figure 1d), it is obvious that massive fine Ni particles were uniformly dispersed on the NiFe_2_O_4_ substrate and acted as catalytic activation sites and could be sufficient to start the electroless Ni-P plating. The Ni concentration at the enrichment zone was determined by EDS spectra to be 89.38 wt.%.

### 3.2. Electroless Ni-P Plating

**Figure 2:**
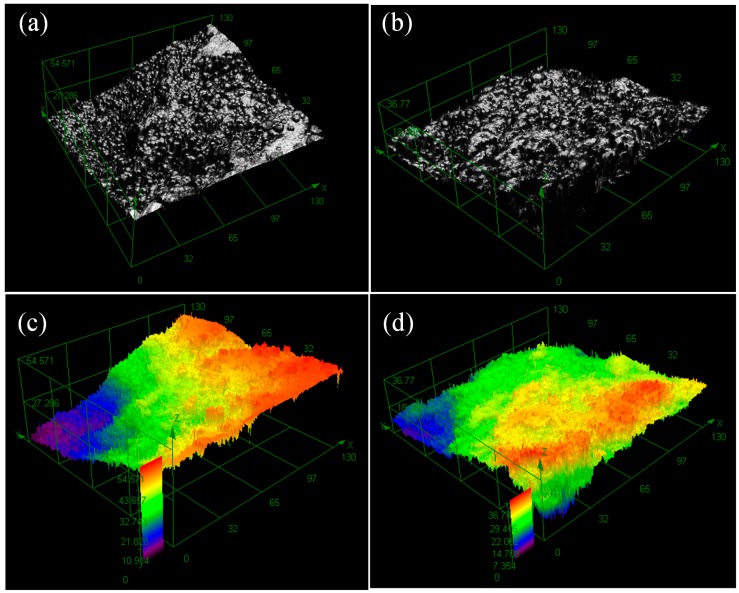
3D measuring laser microscope images of the surfaces of coatings prepared by these two pretreatments: (**a**) 3D brightness image and (**c**) 3D height image of sensitization-activation pretreatment; (**b**) 3D brightness image and (**d**) 3D height image of Pd-free activation pretreatment.

To compare these two pretreatments, NiFe_2_O_4_ particles undergoing the two methods were electrolessly plated under the same conditions. Figure 2 shows the 3D measuring laser microscope images. At the same plating layer area of 130 × 130 μm^2^, we found the height difference in Figure 2c was larger than that in Figure 2d (54.57 μm vs. 36.77 μm), as the plating layer of the sensitized particles was slope-shaped, while that of the Pd-free treated particles much gentler. The roughness (Sa) of the plating layer of the sensitized particles was larger than that of the Pd-free treated particles (4.21 μm vs. 3.18 μm).

**Figure 3:**
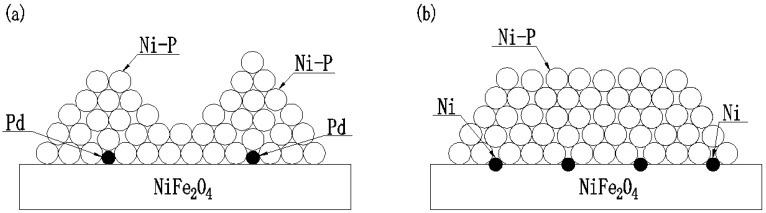
Schematic diagrams of electroless plating process by (**a**) sensitization-activation pretreatment and (**b**) Pd-free activation pretreatment.

The differences between Figure 2a,b are mainly attributed to the numbers of Pd and Ni particles per unit area of the substrate. Compared with the Pd active species derived from the sensitization-activation pretreatment (Figure 1a), more Ni active species are uniformly dispersed on Ni-NiFe_2_O_4_ particles (Figure 1b). The reason may be that less Sn^2+^ was left on the surface of NiFe_2_O_4_ particle, leading to little Pd adsorption on the particle surface activation, while the existence of Pd was necessary for Ni deposition. The grains grew along the three-dimensional directions in the electroless plating [32], and the mechanism underlying the effect of active point distribution on the plating morphology is shown in Figure 3. When the active species were sparse, the grains were prone to crystallization into Volmer-Weber growth (Figure 3a), and other wise, smooth surfaces were formed (Figure 3b).

**Figure 4:**
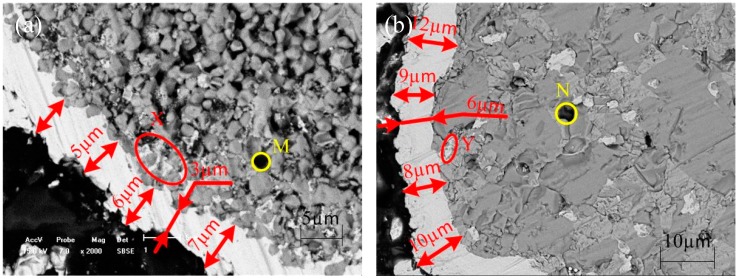
SEM images of Ni-P coatings on NiFe_2_O_4_ electroless plated (**a**) with sensitization-activation and (**b**) Pd-free pretreatment.

Under the same process conditions, the coating layers of NiFe_2_O_4_ particles after electroless plating via Pd-free activation pretreatment are lightly thicker than these via sensitization-activation pretreatment (about 9 μm vs. 5 μm; Figure 4a,b). These results also confirm the new Pd-free pretreatment method could endow the matrix particles with stronger auto catalytic ability. Moreover, the particle substrate in Figure 4a contains numerous holes in the zone M, while that in the zone N in Figure 4b has fewer holes. This was because the addition of Ni powder promoted the sintering and made the ceramic phase denser. The addictive Ni of the Y zone in Figure 4b could enhance the matrix density, while the metals in the zone X in Figure 4a are deposited on the surface holes of the metals during the electroless plating, thereby reducing the thickness of metal plating layer in Figure 4a.

### 3.3. Influences of Main Factors on Pd-Free Electroless Plating

To further explore the main influence factors of electroless Ni-P plating bath on *R* and microstructure of coating, a series of experiments were performed by single factor experiment. The electroless Ni-P plating mechanism was considered to interpret the effects of main factors on *R*. In general, the electroless Ni-P plating underwent the following reactions [33]:

(2) $$H_{2}{\mathrm{PO}}_{2}{}^{-}+H_{2}O\to {\mathrm{HPO}}_{3}{}^{2-}+{2H}_{\mathrm{ad}}+H^{+}$$

(3) $${\mathrm{Ni}}^{2+}+{2H}_{\mathrm{ad}}\to {\mathrm{Ni}+2H}^{+}$$

(4) $$H_{2}{\mathrm{PO}}^{2-}+H_{\mathrm{ad}}\to H_{2}O+{2\mathrm{OH}}^{-}+P$$

(5) $${2H}_{\mathrm{ad}}\to H_{2}↑$$

**Figure 5:**
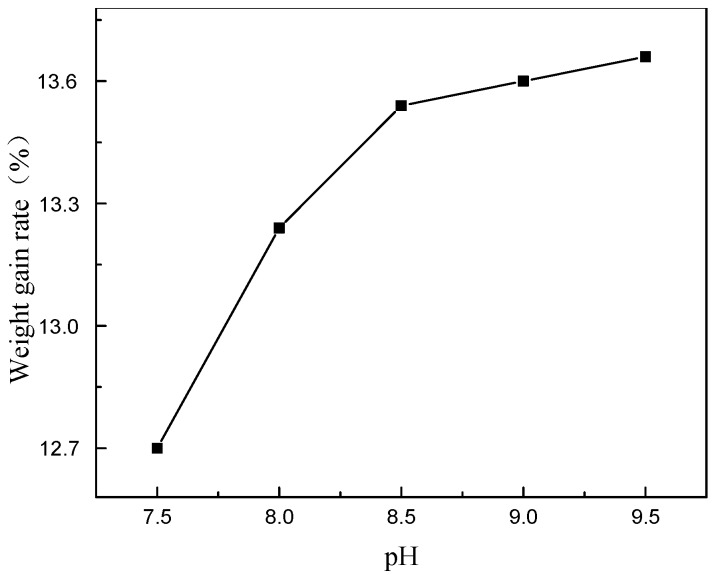
Effect of pH value on the weight gain rate.

Firstly, when pH was changed with in 7.5~9.5, *R* increased, but when the pH exceeded 9.0, the plating solution became less stable and black sediments were formed at the bottom of the bath (Figure 5). These phenomena are probably attributed to the byproduct of electroless plating. According to reaction (2), phosphate anion is generated by the oxidation of hypophosphite. As the reaction proceeds, phosphate steadily accumulates in the plating bath. When the concentration increases to some extent, phosphate begins to complex with Ni^2+^.

Therefore, the experimental results suggest an optimum pH of 9.0.

**Figure 6:**
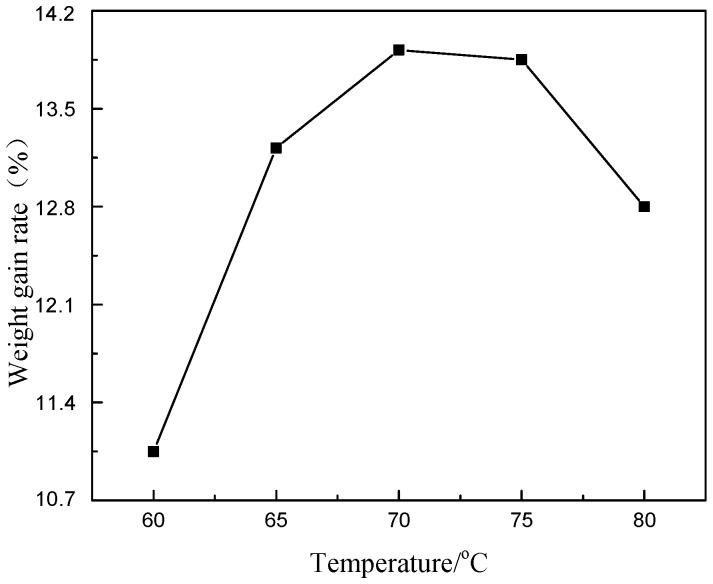
Effect of temperature on the weight gain rate.

Then the effect of bath temperature on *R* was investigated (Figure 6). With the rise of bath temperature, *R* increased first, maximized at 70 °C and then decreased. Electroless Ni-P plating is an endothermic reaction that needs to absorb energy from the surrounding. Indeed, all practical baths should operate at ≥60 °C. Though temperature rise contributed to improving *R*, too high temperature would lead to too fast deposition, which loosened the plating layers and caused the fall-off of the plating layers due to the stirring of the plating liquids. As a result, the concentration of free Ni in the plating liquids increased, which reduced the quality of the plating layers. In this case, the optimal temperature is 70 °C.

Moreover, the plating layer formed at 80 °C was dark grey, and the particles became darker with the increase of bath temperature. Thus, the color changes indicate a different deposition process of Ni-P. To clarify the underlying reasons, the microstructures of the products formed at 70 °C and 80 °C were further analyzed.

**Figure 7:**
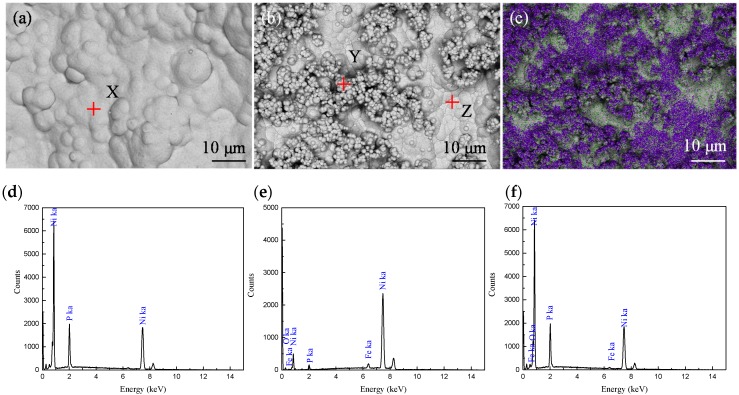
The SEM morphologies of the coating at (**a**) 70 °C and (**b**) 80 °C, EDS results of the coating, on X (**d**) and Y (**e**), Z (**f**), and mapping of elements of P in (**c**).

Figure 7 shows the SEM images of the Ni-P-coated NiFe_2_O_4_ particles at 70 °C (Figure 7a) and 80 °C (Figure 7b). The coating formed at 70 °C was constituted with agglomerated nodules and was smooth and uniform (Figure 7a). In the coating formed at 80 °C, however, a lot of microspheres deposited and already agglomerated on the nodules of the deposited layer (Figure 7b). EDS hierarchical scan image in Figure 7c obtained from P in 7b shows P is enriched in the of microsphere gathering zone of Figure 7b.

The compositions of the nodules and the microsphere formed on NiFe_2_O_4_ particle surface were investigated via EDS (Figure 7d–f). As electroless plating proceeded at 70 °C, the X site on the EDS image only contained Ni (89.83 wt.%) and P (10.17 wt.%), as the surface was fully covered by deposited Ni-P coating (Figure 7d). As electroless plating proceeded at 80 °C, the signals at points Y and Z of Fe and O were weakened (Figure 7e,f), indicating the coating formed on the surface of NiFe_2_O_4_ particles was thin. At point Z compared with point X which were both nodules, the content of P element was far less (0.7% vs. 10.17%) and the content of Ni element was far more (96.72% vs. 89.83%). However, at point Y which was in the microsphere, the content of P element was the highest (13.24%) and the content of Ni element was the least (85.04%). This observation is in good agreement with Figure 7c that P is enriched in the microspheres.

**Figure 8:**
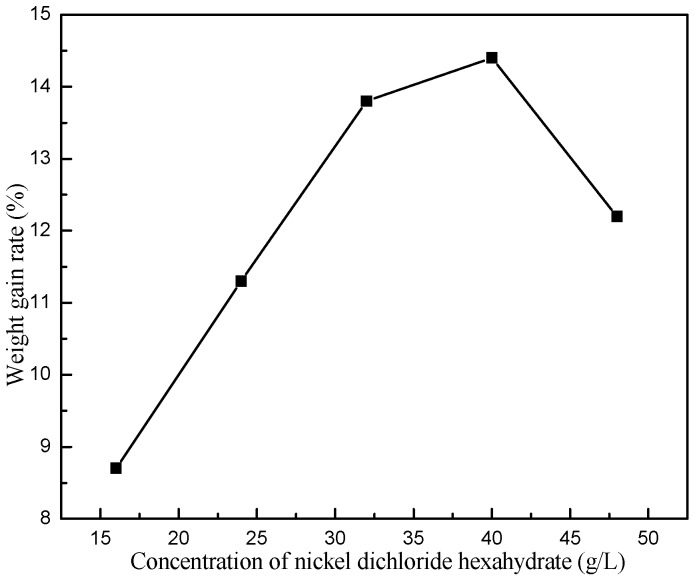
Effect of NiCl_2_·6H_2_O concentration on the weight gain rate.

Finally, the influence of Ni^2+^ concentration on *R* was investigated. Clearly, *R* rose linearly with the increase of Ni^2+^ concentration in the range from 16 to 32 g/L (Figure 8), which was because Ni^2+^ was reduced by H_2_PO^2−^ according to reactions (2) and (3). However, *R* slightly rose as Ni^2+^ concentration increased beyond the level of 40 g/L. This was probably because the complex formation between complexing agents and nickel ions maintained the dissociated nickel ion concentration appreciably constant at a higher Ni^2+^ concentration in the electroless Ni-P plating bath. Thus, the increase of Ni^2+^ concentration at a constant complexing agent concentration did not immediately increase the amount of free Ni^2+^ in the bath. Further increase in Ni^2+^ concentration at a high range (≥40 g/L), led to obvious decrease of *R*. From the economic perspective, higher Ni^2+^ concentration of the electroless Ni-P plating bath is not recommended. Hence, these results verify the optimized concentration of NiCl_2_·6H_2_O is around 32 g/L.

Based on the above results and discussion, a mechanism of the proposed electroless Ni-P plating at 80 °C is as follows: reaction (3) is dominant at the beginning of the electroless Ni-P plating, in which a mass of Ni particles and a few P particles are deposited to form nodules. Then reaction (4) becomes dominant, in which a mass of P particles and a few Ni particles are deposited to form microspheres.

## 4. Conclusions

A novel Pd-free activation pretreatment for Ni electroless plating on NiFe_2_O_4_ was achieved by fixing Ni particles as catalysts on the NiFe_2_O_4_ substrate. With this pretreatment method, a glossy and smooth Ni-P plating layer was formed from an electroless Ni-P plating bath. This environmentally friendly activation process is contributed to reducing both capital and operational costs in large-scale manufacturing. Experiments about the influences of main factors in the electroless Ni-P plating bath on *R* and microstructure of coatings show the optimal NiCl_2_·6H_2_O content, pH and temperature are 32 g/L, 9.0 and 70 °C, respectively.

## Figures and Tables

**Table 1:** Technological conditions of electroless plating.

	Electroless Plating Bath				
Reagents	NiCl_2_·6H_2_O	NaH_2_PO_2_·H_2_O	C_6_H_5_Na_3_O_7_·2H_2_O	C_3_H_6_O_3_	CH_3_COONa
Concentration	16–48 g/L	28 g/L	25 g/L	20 mL/L	15 g/L
Process time	70 min				
Temperature	60–80 °C				
pH	7.5–9.5				
Stirring speed	250 rpm				

**Table 2:** Size characteristics of NiFe_2_O_4_ and Ni-NiFe_2_O_4_ particles.

Characteristics	NiFe_2_O_4_	Ni-NiFe_2_O_4_
D10	152 μm	153 μm
D50	236 μm	234 μm
D90	350 μm	346 μm
Specific surface area	0.008 m^2^/g	0.008 m^2^/g
